# Epidemiology and outcome of cervical cancer in national institute of Morocco

**DOI:** 10.1186/s12905-016-0342-2

**Published:** 2016-09-13

**Authors:** Sanaa Elmajjaoui, Nabil Ismaili, Hanane El Kacemi, Tayeb Kebdani, Hassan Sifat, Noureddine Benjaafar

**Affiliations:** 1 Department of Radiotherapy, National Institute of Oncology, Mohammed V University, Rabat, Morocco; 2 Medical Oncology, Cheikh Khalifa Ibn Zaid Hospital, Université Mohammed VI des Sciences de la Santé, Casablanca, Morocco; 3 Department of Radiotherapy, Mohammed V Hospital, Mohammed V University, Rabat, Morocco

**Keywords:** Cervical cancer, Epidemiology, Treatments, Outcomes

## Abstract

**Background:**

On behalf of the medical staff of the National Institute of Oncology of Rabat, we conducted a retrospective study to report epidemiology and 5-year outcomes of cervical carcinoma in Moroccan women.

**Methods:**

We reviewed all women diagnosed with invasive cervical carcinoma in our institute between January 2006 and December 2006. Outcomes and prognoses are analyzed in patients who received at least one treatment.

**Results:**

The analysis included 646 women. Median age was 50 years (23–85 years). Bleeding was the most frequent symptom (95 %). The most predominant histology was squamous cell carcinoma (94 %). The majority of patients were diagnosed at locally advanced stages (88 %). Among patients who received treatment (*n* = 550), the management was based on concurrent chemoradiotherapy in 69.7 % of cases. The median duration of follow-up was 60 months (range 2–78 months). Overall survival, progression free survival, and locoregional recurrence free survival were 63.2, 60.7 and 79.1 % respectively. Significant poor prognostic factors in univariate analysis included stage, tumor size, lymph node involvement, anemia and absence of response to radiotherapy. The prognostic significance of response to radiotherapy and stage were retained in multivariate analysis.

**Conclusion:**

Cervical cancer in our Institute is diagnosed at locally advanced stages. Two third of patients were treated by concurrent chemoradiotherapy. Outcome of Moroccan patients are comparable to that of western countries. Significant prognostic factors were stage, tumor size, lymph node involvement, anemia, and response to radiotherapy. The way to reduce the global burden of cervical cancer in our country continues to be the development of vaccination and screening programs.

**Electronic supplementary material:**

The online version of this article (doi:10.1186/s12905-016-0342-2) contains supplementary material, which is available to authorized users.

## Background

Cervical cancer is the third most common cancer and the fourth leading cause of cancer death in females in the world, accounting for 9 % (529,800) of the new cancer cases and 8 % (275,100) of the cancer deaths among females in 2008 [1, 2]. Its incidence has remarkably decreased in western countries through organized screening programs [3]. However, in Africa, in general, and in Morocco especially, cervical cancer represents a major health problem and the leading cause of cancer death in women because of the diagnosis at advanced stages [1]. In Moroccan women, cervical cancer is the second most common cancer after breast cancer [4]. The incidences of cervical cancer in Casablanca and Rabat were 14.4/100000 (new cases per 100000 women) and 13/100000, respectively.

The aim of our study was to assessing the current outcomes after a diagnosis of cervical cancer since 2005 when campaigns to improve prevention and treatment were launched by the efforts of the government and of the Lalla Salma Foundation Prevention and Treatment of Cancers. In a previous investigation of cervical cancer in young Moroccan women, treated between 1980 and 1990,overall survival rate was only 41 % at 5 years [5]. Our hypothesis is that the outcomes of cervical cancer were improved as compared to previous data.

## Methods

### Study design

We retrospectively investigated women of all ages withsquamous-cell carcinoma, adenocarcinoma, or adenosquamous carcinoma of the cervix of International Federation of Gynecology and Obstetrics (FIGO 2002) stages I, stage II, stage III of stage IV (Additional file 1, page 1). Women were excluded from the study if they met any of the following criteria: lack of pathological confirmation of invasive carcinoma; and in situ cervical cancers.

All women diagnosed with invasive cervical carcinoma, between January 2006 and December 2006, were included. In 2006, Morocco had only two cancer centers, the most important was National Institute of Oncology of Rabat which includes mainly patients coming from northern and eastern regions of Morocco, the second was Casablanca cancer center which include mainly patients coming from southern regions of Morocco. So the population included in our series is representative of the general population of our country.

The ethical approval for the study was obtained from our ethics committee; the National Institute of Oncology ethics committee. Written informed consent was not required, however all patients provided there oral consent. The ethics committee approved the verbal informed consent procedure.

Patient’s medical records were reviewed for investigations of demographic characteristics, clinical signs, clinical sage, histological results, and outcomes. Biological work-ups were analyzed. Radiological reports were reviewed to determine the stage of the disease caused by the invasive carcinoma. Data concerning treatment modalities, CCRT, RT, Surgery, adjuvant treatments (CCRT, RT, brachytherapy), preoperative treatments, and palliative treatments (radiotherapy and chemotherapy), were collected and analyzed.

During radiotherapy treatment, patients were evaluated weekly by clinical examination. During concurrent chemo-radiotherapy, patients were evaluated weekly by clinical examination, a complete blood count and serum levels of creatinine. Patients had a pelvic examination under anesthesia at the time of each intra-cavity treatment (brachytherapy). During palliative, neo-adjuvant or adjuvant chemotherapy, patients were evaluated every 3 weekly by clinical examination, complete blood count and serum levels of creatinine. Once the treatment ended, patients were evaluated every 3 months for the first 2 years, every 6 months during the third, fourth and fifth years, and then annually. Disease status was assessed by history taking, physical examination, and appropriate laboratory and radiologic tests. Safety was assessed at the time of each evaluation with the use Cooperative Group Common Toxicity Criteria.

We analyzed the outcome of patients treated in our institute and the impact of certain prognostic factors on survival: stage of the disease, tumor size, lymph node involvement, anemia, and response to radiotherapy. These factors were chosen because they affect the survival of patients diagnosed with cervical carcinoma.

### Statistical analysis

Loco-regional recurrence free survival (LRRFS) (locoregional events occurred in the cervix and/or regional lymph nodes, with or without concurrent metastatic recurrence) was calculated from the date of diagnosis (biopsy) to the date of first documented locoregional relapse. Progression free survival (PFS) was calculated from the date of diagnosis (biopsy) tothe date of the first physical or radiographic evidence of disease progression, death or the last follow-up visit. Overall survival (OS) was calculated from the date of diagnosis (biopsy) to the date of death or the last follow-up visit. Kaplan-Meier method was used to estimate the rates of 5 years LRRFS, PFS, and OS. Log-rank-test was used to evaluate the differences between the groups [6].

Univariate and multivariate Cox proportional hazard regression models analyses were used to evaluate the relationship between survival (LRRFS, PFS, and OS), and prognostic factors (age, histology, stage of disease, lymph node involvement, tumor volume, response to treatment, and anemia) [7]. Candidate prognostic factors for LRRFS, PFS, and OS, with a 0.5 level of significance in univariate analysis were entered in a multivariate Cox model. Statistical evaluation was carried out using SPSS 17.0 statistical software.

Patients lost to follow up before to receive at least one treatment modality [CCRT, exclusive RT, Surgery, brachytherapy, and palliative treatments (radiotherapy and chemotherapy)] were excluded from efficacy and safety analyses (96 women). Patients lost to follow up during the treatment or the follow up period were censored.

### Study endpoints

The study endpoints include: the analysis of epidemiological characteristics, evaluation of 5-year outcomes (loco-regional control, progression free survival, and overall survival), and analysis of prognostic factors.

Treatment of cancers in Morocco has significantly evolved the last decade through the efforts of the government and of the Lalla Salma Foundation Prevention and Treatment of Cancers. Our hypothesis is that the outcomes of patients with cervical cancer were improved.

## Results

From January 2006 to December 2006, 646 patients were enrolled. All of them were included in the epidemiological analyzes of demographic and clinical characteristics. Patients who did not receive any treatment were excluded from efficacy and safety analyses.

### Patient and tumor characteristics (Tables **1** and **2**)

**Table 1:** Demographic characteristics for all patients included (*n* = 646)

Characteristics	Patients number (%)
Age	
Median	50 years
Range	23–85 years
Age intervals	
15–24 years	1 (0.2 %)
25–34 years	41 (6.3 %)
35–44 years	172 (26.6 %)
45–54 years	197 (30.5 %)
55–64 years	136 (21.1 %)
65–74 years	73 (11.3 %)
> 75 years	26 (4 %)
Menopausalstatus	
Menopausal	343 (53 %)
Premenopausal	303 (47 %)
Marital status	
Single	33 (5 %)
Maried	517 (80 %)
ND	96 (15 %)
Age at first marriage	
< 20 years	381 (59 %)
> 20 years	265 (41 %)
Number of parities	
0	27 (4.2 %)
< 4	178 (27.5 %)
> =4	407 (63 %)
ND	34 (5.3 %)

*Abreviations*: *ND* not defined

**Table 2:** Findings on clinical examination

Clinical examination	Number of patients (%)
Performance status	
PS 1	530 (82 %)
PS 2	110 (17 %)
PS 3	6 (1 %)
Tumor size	
< 4 cm	72 (11 %)
≥ 4 cm	574 (89 %)
State of the vagina	
Not invaded	133 (20.6 %)
Invaded	513 (79.4 %)
Upper third	263 (40.7 %)
Middle third	196 (30.3 %)
Lower third	54 (8.4 %)
State of parameters	
Not invaded	137 (21.2 %)
Invaded	509 (78.8 %)
Proximal	86 (13.3 %)
Distal	161 (25 %)
Until the wall	262 (40.5 %)
Clinical stage	
IA	1 (0.1 %)
IB1	63 (9.8 %)
IB2	24 (3.7 %)
IIA	40 (6.3 %)
IIB	235 (36.5 %)
IIIA	3 (0.4 %)
IIIB	252 (39 %)
IVA	13 (2 %)
IVB	14 (2.2 %)

Median age was 50 years (range 23–85 years). Median duration of evolution of symptoms (from onset of symptoms to consultation at our center) was 5 months (range: 1–48 months). Bleeding was the most common symptom showed in 95 % of cases, followed by leukorrhea (50.2 %).

Squamous cell carcinoma (SCC) histology was the most common pathological type and was reported in 94 % of cases followed by adenocarcinoma in 5.5 % of cases. Only 0.5 % of cases have adenosquamous carcinoma. Tumor size was equal or greater than 4 cm in 81 % of cases with a median tumor size of 7 cm. Patients have locally advanced stages (IB2, II, III and IV), according to FIGO 2002 staging system, in 88 % of the cases. External iliac, internal iliac and para-aortic lymph nodes were observed in 96 cases (15 %), 70 cases (10.8 %), and 45 cases (7 %), respectively, and they were most often associated. Only fourteen patients were metastatic at presentation (2.2 %).

### Treatment modalities (Fig. 1) (*n* = 550)

**Fig. 1:**
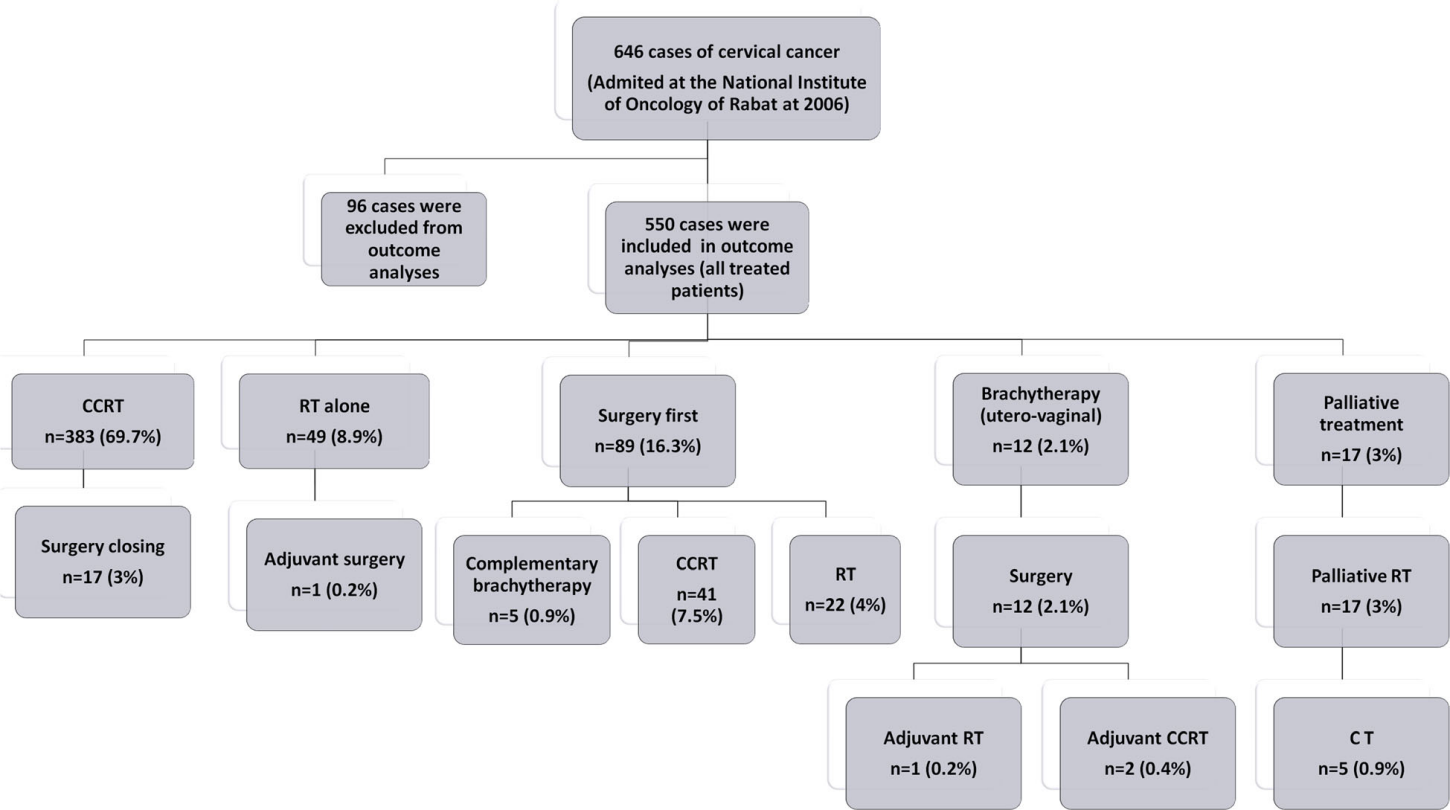
Treatments modalities

Among 646 patients, the treatment has not been done in 96 cases (14.6 %). These patients were lost to follow-up before the initiation of management and were excluded from efficacy analyses. This is largely explained by the socio-economic level, often poor in our population.

Three hundred eighty three patients (69.7 %) underwent curative **CCRT**. Among these patients, 17 received adjuvant surgery. Seven cases have complete pathological response in surgical specimens. **Exclusive radiotherapy** was indicated in 49 patients (8.9 %), only one patient received adjuvant surgery (staged IIA with a size of 3.5 cm). After EBRT (CCRT or exclusive RT, *n* = 432), 310 (72 %) patients received utero-vaginal brachytherapy, 56 cases (13 %) received external four fields RT at a dose of 24 Gy and 18 (4 %) received adjuvant surgery. Unfortunately, 48 patients (11 %) did not receive complementary treatment. Three hundred thirteen patients presented with stage IIB distal or IIIB, 213 among them received a complementary parametrial dose (9 Gy [3 × 3Gy] or 10 Gy [5 × 2 Gy]), 46 patients received four additional radiation fields at a dose of 24 Gy (2 Gy per fraction), and 54 patients did not receive complementary parametrial dose. Among 137 patients who had suspicious pelvic lymph node on imaging, 102 received complementary dose of RT, while 35 patients did not receive lymph node complementary dose. Nine of 10 patients who have para-aortic lymph node received a complementary dose of 10–10.8 Gy (1.8-2Gy per fraction). A total of 81 patients did not complete their treatment (lost to follow up).

**Surgery** was the initial treatment of 89 cases (16.3 %), 68 of them (85 %) received adjuvant treatment: CCRT in 41 cases, RT in 22 cases, and brachytherapy in 5 cases. Twelve cases received **preoperative brachytherapy** followed by surgery (2.1 %), 1 of them received adjuvant RT, and 2 patient received adjuvant CCRT.

**Neoadjuvant chemotherapy** was not a standard treatment in our institution, and was indicated in only 20 patients. **Adjuvant chemotherapy** was administered in 2 patients; in one case after surgery (stage IB1), and in a second case after RT (stage IIA).

**Palliatives treatments** were administered in 17 patients and were based on palliative RT in 12 cases and palliative RT plus palliative CT in 5 cases.

### Toxicities

Table 3 shows the frequency of acute toxicities potentially associated with CCRT. Gade 3 and 4 anemia and neutropenia were observed in 8.3 and 4 %, respectively. Renal failure was showed in only 0.5 % of cases.

**Table 3:** Acute toxicities in patients treated with CCRT (*n* = 425)

	Number of patients	Percent
Anemia		
Grade 1	113	26.6 %
Grade 2	78	18.3 %
Grade 3	31	7.3 %
Grade 4	4	1 %
Neutropenia		
Grade 1	51	12 %
Grade 2	21	5 %
Grade 3	28	6.5 %
Grade 4	2	0.5 %
Renal failure	2	0.5 %

### Outcomes

Patients who received at least one treatment (*n* = 550) were included in the efficacy analysis.

#### Response to treatments

Table 4 shows the response to treatments at 3-months.

**Table 4:** Response to treatments at 3-months

Treatment	Response
Surgery first (*n* = 89)	R0 resection (93.2 %)
CCRT (*n* = 363)	ORR (91 %)
	CR (65 %)
	PR (26 %)
	PD (6.5 %)
	Unknown response (2.5 %)
RT (*n* = 48)	ORR (81 %)
	CR (79 %)
	PR (2 %)
	PD (19 %)

*CCRT* Concurrent chemoradiotherapy, *ORR* Objective response rate, *CR* complete response, *PR* Partialresponse, *PD* Progression disease, *RT* radiotherapy

#### Overall survival

**Fig. 2:**
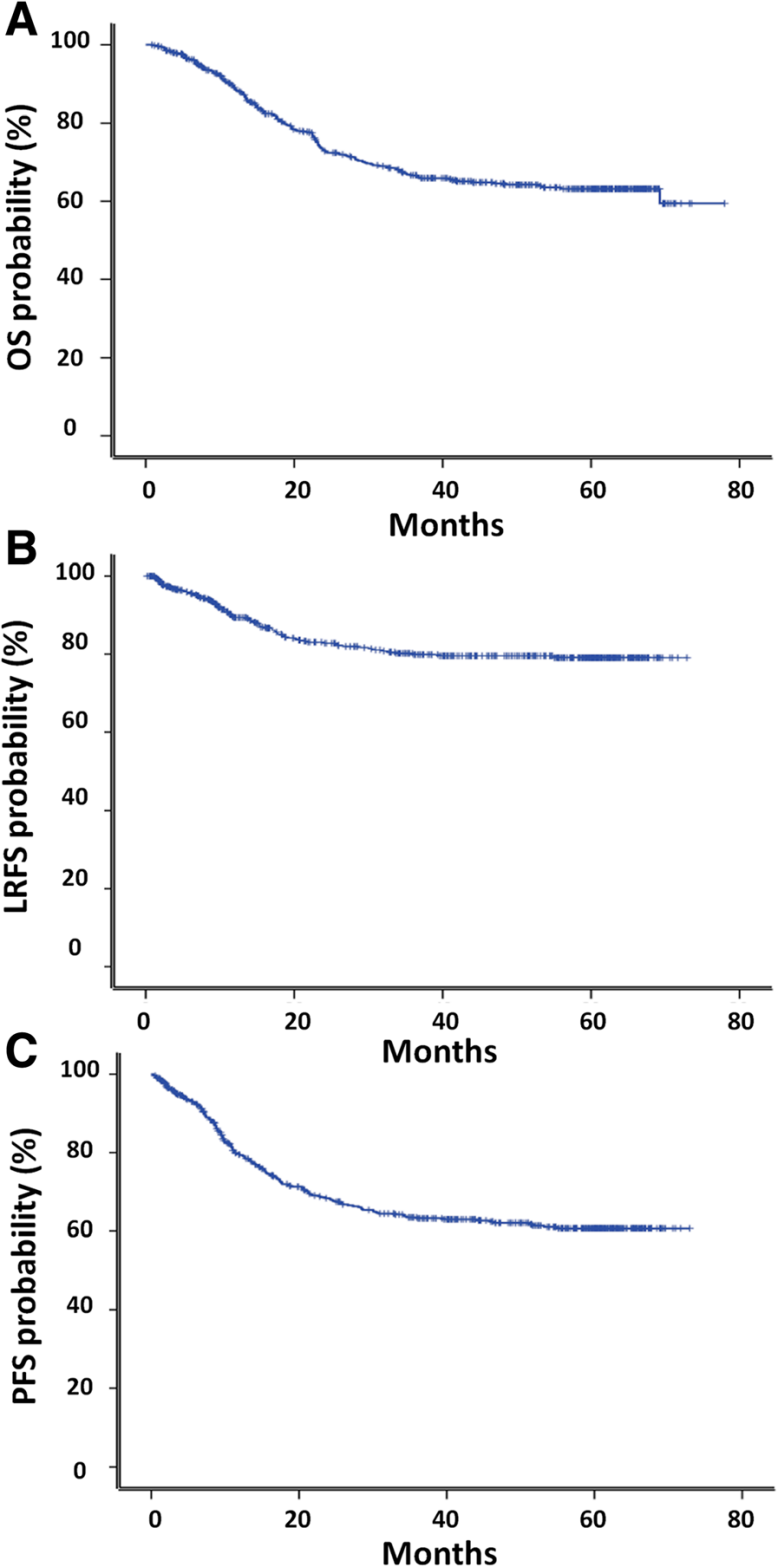
Survival curves. Figure 1a: Overall survival; Fig. 1b: Locoregional recurrence free survival; Fig. 1c: Progression free survival

At 60 months median follow up, 160 deaths had been reported. The probability of 5 year overall survival was 63.2 % (Fig. 2a).

#### Progression free survival

At 60 months median follow-up, 175 patients developed recurrence, local recurrence in 22.7 % of the cases (*n* = 125) and metastatic recurrence in 13.8 % of the cases (*n* = 74) (bone metastases in 26 cases [35.1 %], lung in 13 cases [17.5 %], peritoneal in 10 cases [13.5 %] and hepatic in 9 cases [12.2 %]). The probabilities of LRRFS and PFS at 5 years were 79.1, and 60.7 %, respectively (Fig. 2b and c).

#### Locoregional recurrences

Locoregional recurrences occurred in 22.7 % of the cases, and metastasis recurrence occurred in 13.8 % of the cases. Locoregional recurrences were associated with metastatic evolution in 4.3 % of cases. 82.9 % of relapses occurred in the first 2 years after treatment and 63.4 % of recurrences are centro-pelvic.

### Prognostic factors

**Fig. 3:**
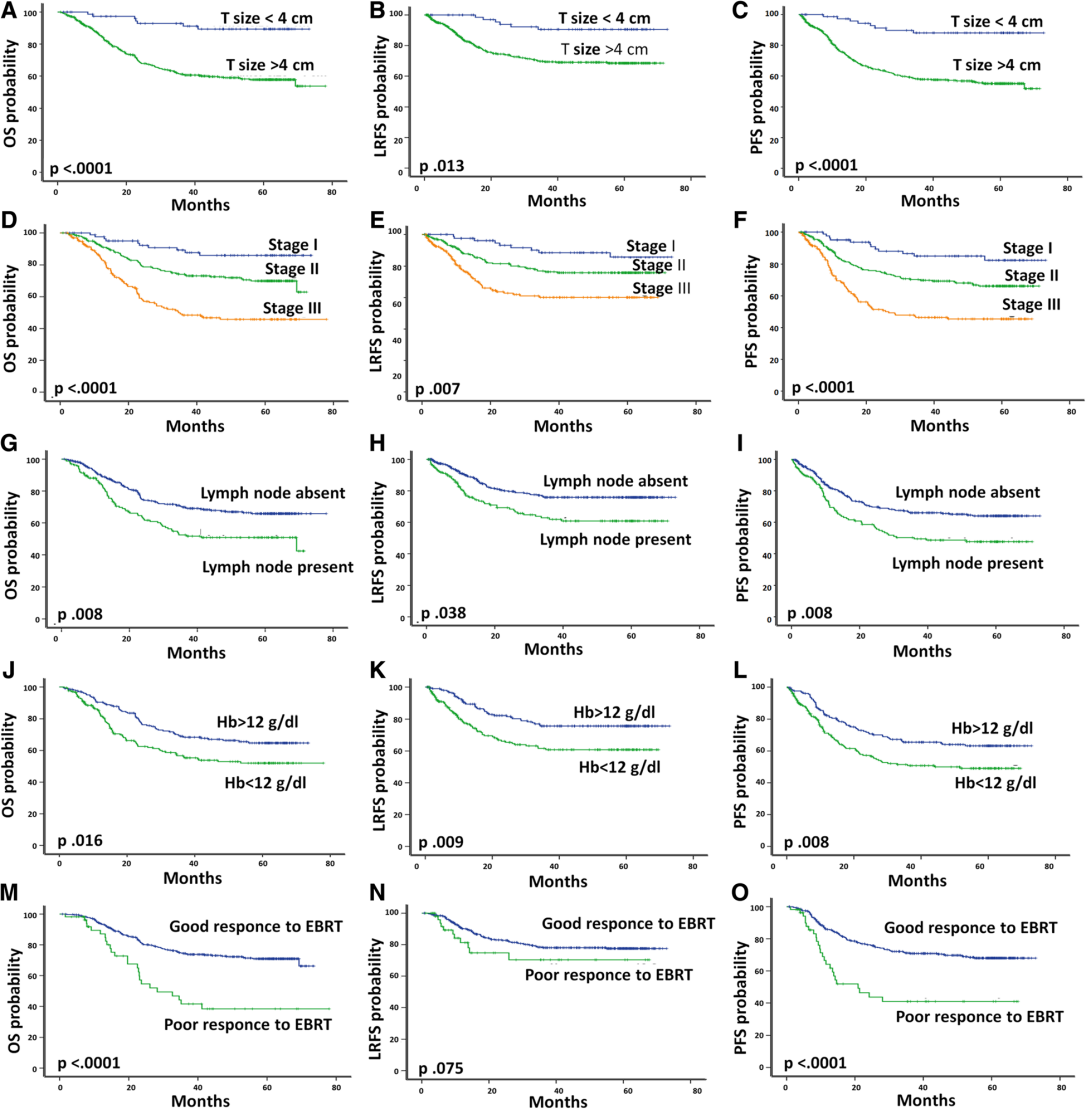
Prognostic factors. Overall survival, Loco regional recurrence free survival, and progression free survival curves according to prognostic factors. (4**a**, 4**b**, 4**c**): Tumor size; (4**d**, 4**e**, 4**f**): Clinical stage; (4 **g**, 4 **h**, 4**i**): Lymph node involvement; (4**j**, 4 **k**, 4 **l**): Hemoglobin level less than 12 g/dl during radiotherapy; (3 M, 3 N, 3O): Tumor response to radiotherapy

In univariate analysis (Fig. 3 [a to o]), we identified five poor prognostic factors with a statistically significant impact on the disease: tumor size, clinical stage, lymph node involvement, hemoglobin level less than 12 g/dl during RT, and absence of response to radiotherapy (46 Gy). Tumor size (greater than 4 cm) was a poor prognostic factor that affected significantly OS (*p* < 0.0001), PFS (p <0.0001) and LRRFS (*p* = 0.013). Clinical stage: Clinical stage affected significantly the prognosis of our patients: OS (*p* < 0.0001), PFS (*p* < 0.0001) and LRRFS (*p* = 0.007). Lymph node involvement (suspected on imaging) was a statistically poor prognostic factor influencing OS (*p* = 0.008), PFS (*p* = 0.038) and LRRFS (*p* = 0.008). Anemia during RT affected all parameters of survival: OS (*p* = 0.016), PFS (*p* = 0.008) and LRRFS (*p* = 0.009). Absence of response after a dose of 46 Gy of RT influencing OS (*p* < 0.0001), PFS (*p* <0.0001) and LRRFS (*p* = 0.075).

Multivariate analysis performed by using cox proportional hazard model showed that response to RT and clinical stage are two independent factors influencing outcome of patients with cervical carcinoma in our population. **Absence of response to RT** was a prognostic factor influencing significantly and independently PFS (*p* = 0.001) and OS (*p* = 0.001). **Clinical stage** was a prognostic factor affecting LRRFS (*p* = 0.09), PFS (*p* = 0.033) and OS (*p* = 0.012).

## Discussion

We conducted a retrospecive study to evaluate the epidemiology and outcome of cervical carcinoma in Morocco. To our best knowledge, our study was the first published study evaluating the epidemiological characteristics and prognostic of cervical cancer in our country. We found the predominance of locally advanced stages, so the diagnosis was most often made at advanced stages in 88 % of cases (IB2, II, III and IV in 3.7 %, 6.3 %, 36.9 %, 39 %, 2 % respectively). The majority of patients (69.7 %) received CCRT. At 5 years, PFS and OS were 63.2 and 60.7 % respectively. Outcomes of our patients with cervical carcinoma are influenced by response to RT and clinical stage who are two independent factors retained in multivariate analysis. After RT, patients with clinical response to radiotherapy had a higher rate of PFS and OS.

One potential drawback of our study was the high number of lost to follow up patients before management. The inclusion of these women may have affected the overall survival from cervical cancer. This is due to low socio-economic level of most cases included in our institute. These patients have been excluded from the efficacy analyses. In addition, we showed a high rate of locoregional recurrences (22.7 %) which may be due mainly to the delay of diagnosis, and secondarily due to the quality of treatments received; surgery, lymph node dissection, radiotherapy, spreading of radiation which is very large in most cases (median 84 days), and radiation - brachytherapy period which exceeded 6 weeks in all cases. Among the strength of our study : the large sample size and the standardized treatments available in our institute (surgery and or radiotherapy with or withouth chemotherapy and brachytherapy).

Our results have shown that cervical cancer in Morocco is diagnosed at advanced stages in 88 % of the cases. Several retrospective studies have shown similar findings. In South American population (*n* = 190), stage distribution was the following: II and IIIB in 93.7 % of cases. [8]. In the Korean series [9], we noted 43.8 % of locally advanced stages IB2-IV. However, in Europe, cervical cancer, was diagnosed mainly at localized stages IB; 73.4 % in France [10]. In an Asiatic series from investigator in Beijing (*n* = 1399) [11], there is a predominance of localized stage I in 57.1 %. The discrepancy noted in the results of different series is due to socio-economic level on one hand and practice in another hand. Indeed, in industrialized countries screening policy exists, the frequency of advanced stages is small compared to that of precancerous lesions and localized forms. While in developing countries, cervical cancer is often discovered at advanced stages due to the **lack** of screening policy.

Outcomes of locally advanced tumors of cervix are improved since CCRT has become the standard of care for these stages, based on the report of several randomized clinical trials and metaanalyses [12]. This therapeutic modality is superior to RT alone in the rate of local control, survival without metastatic recurrence, PFS and OS. In our series, the majority of patients (*n* = 69.7 %) received CCRT.

Five year OS of 63.2 % among patients in our series was much better than that of patients treated in other part of Africa (sub-Saharan Africa); for example, 2 year overall survival observed among patients in a recent Kenyan series was less than 20 % [13]. In addition, the 5 years OS in our study was comparable to that observed among patients in certain developed countries; in Canada 5 years survivals varied from 54 to 67 % [14]; in England population, 5-year survival was 69.8 % [15].

In a serie of cervical cancer in young Moroccan women, treated between 1980 and 1990, overall survival rate was only 41 % at 5 years [5]. We can conclude that prognosis of our population has been improved since the date by introduction of CCRT as a standard treatment of advanced stages.

Five prognostic factors in our study population were associated with a statistically significant impact (in univariate analysis) on LRRFS, PFS and OS: tumor size, clinical stage, lymph node involvement, anemia with hemoglobin lower than 12 g/dl during radiotherapy, and tumor response to RT (46 Gy). Significant prognostic factors in multivariate analysis (by Cox proportional hazard model) include response to RT influencing significantly PFS and OS, and clinical stage was a factor influencing LRRFS, PFS and OS. The main prognostic factors were clinical stage, tumor volume, lymph node metastases identified by lymphography and grade of histological differentiation. Clinical stage is clearly the most powerful determinant of outcome in most scientific papers [16–23].

## Conclusions

Cervical cancer is a major health problem in Morocco due to the high incidence and to the frequent diagnosis at locally advanced stages. Therefore, the most used strategy in our center was CCRT. The outcomes of ourpatient has been improvedas compared to previous published Moroccan data in the 1990s. Significant prognostic factors in our study were the stage, and absence of response to EBRT. To reduce the global burden and mortality of cervical cancer, we should inform and educate women about risk factors and prevention, so we should start vaccines (HPV 16–18) to girls before the start of sexual activity and undertake an effective screening programs for women over 30 years old incorporating validated HPV DNA tests. Governments are challenged to firstly establish a policy of systematic screening with the objective of early detection of cervical cancer, and to obtain an acceptable rate of healing.

## Additional file

Additional file 1: STROBE Statement-Checklist of items that should be included in reports of cohort studies. (PDF 18 kb)

## Abbreviations

CCRT: Concurrent chemoradiotherapy
CR: Complete response
FIGO: International federation of gynecology and obstetrics
LRRFS: Locoregional recurrence free survival
OR: Objective response
OS: Overall survival
PFS: Progression free survival
RT: Radiotherapy
SCCS: Quouamous cell carcinoma
